# Optimization of laccase from *Stenotrophomonas maltophilia* E1 by submerge fermentation using coconut husk with its detoxification and biodecolorization ability of synthetic dyes

**DOI:** 10.1186/s40643-023-00703-x

**Published:** 2023-11-13

**Authors:** Yazeed Albulaihed, Mohd Adnan, Arshad Jamal, Mejdi Snoussi, Kartik Patel, Mitesh Patel

**Affiliations:** 1 Department of Biology, College of Science, University of Ha’il, P.O. Box 2440, Ha’il, Saudi Arabia; 2 Biotech Research and Development Lab, Witmans Industries Private Limited, Daman, Bhimpore, 396210 India; 3 Research and Development Cell, Department of Biotechnology, Parul Institute of Applied Sciences, Parul University, Vadodara, 391760 India

**Keywords:** *Stenotrophomonas maltophilia*, Biodecolorization, Detoxification, Synthetic dyes, Phytotoxicity, Laccase

## Abstract

Enzymatic degradation of synthetic dyes holds an immense promise for addressing the environmental concerns associated with the textile and dye industries. This study aimed to isolate bacteria capable of producing laccase enzymes from an anthropogenic environment. Subsequently, viability of utilizing cost-effective agricultural residues as substrates for laccase production was assessed. Response Surface Methodology (RSM) and the One Variable at a Time (OVAT) approach was pursued for the optimization of laccase production, followed by pH and temperature stability, dye degradation and decolorization experiments, toxicological studies on the degraded dye metabolites. In results, laccase-producing bacterial strain was identified as *Stenotrophomonas maltophilia* strain E1 (*S. maltophilia*). Among variety of substrates, coconut husk exhibited optimal efficacy. In a statistical optimization study, it was found that *S. maltophilia* was capable of producing laccase 51.38 IU/mL, i.e., three times higher than the amount of laccase produced by unoptimized medium (16.7 IU/mL), and the enzyme activity was found to be steady at an acidic pH, and a mesophilic temperature range. The laccase obtained from *S. maltophilia* E1 demonstrated proficient dye decolorization capabilities, achieving a notable 92.1% reduction in Malachite green dye coloration at a concentration of 500 ppm. Gas chromatography–mass spectrometry (GC–MS) analysis of the decolorized derivatives of Malachite green revealed a conversion into a distinct compounds. Moreover, after undergoing laccase treatment, Malachite green exhibited decreased phytotoxic effects on *Oryza sativa*, pointing to enzymatic detoxification. Collectively, insights gained from the present study will contribute to the development of efficient enzymatic approaches for addressing the environmental pollution caused by synthetic dyes.

**Graphical Abstract:**

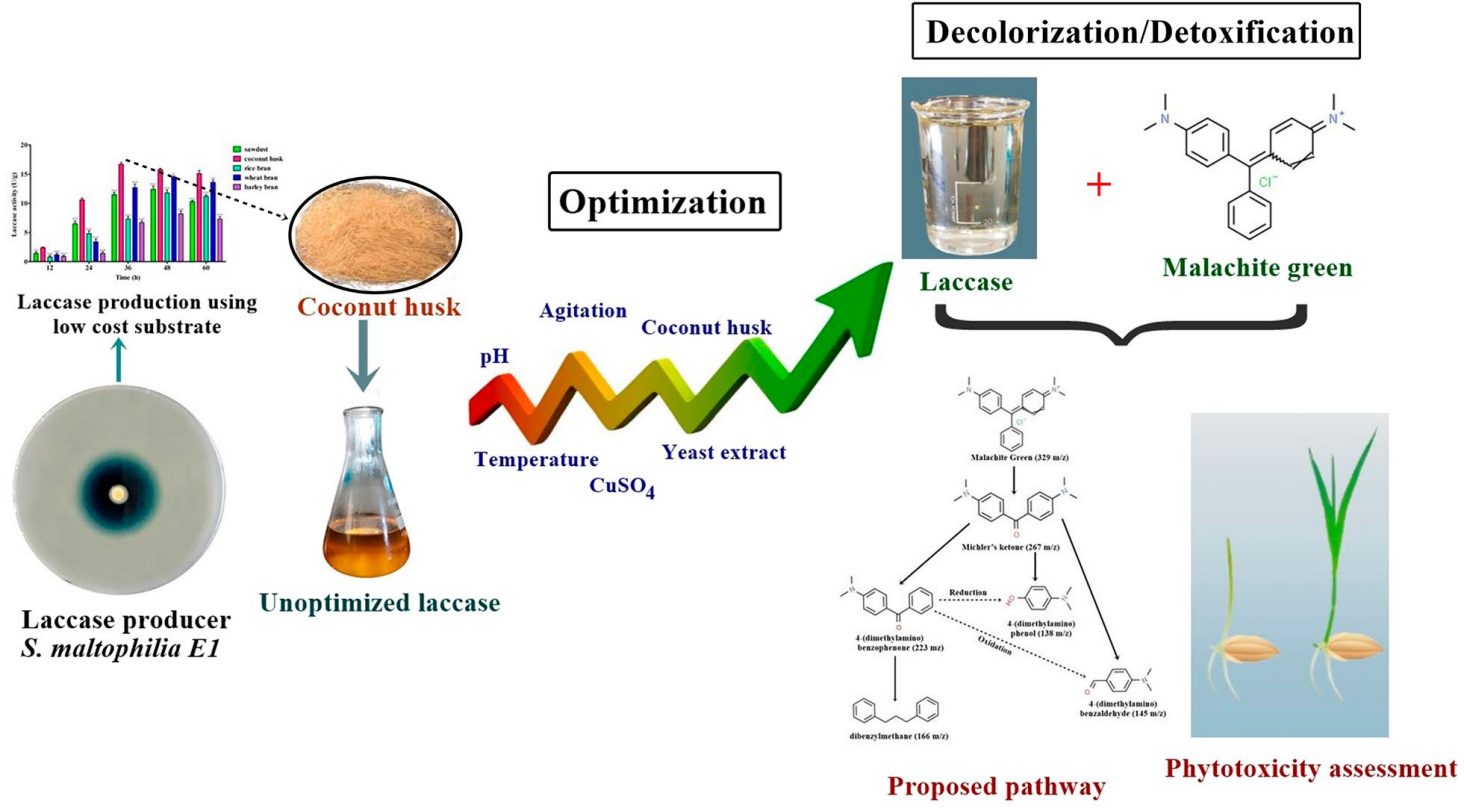

## Introduction

Laccase (EC 1.10.3.2) is a copper rich polyphenol oxidase that catalyzed the oxidation of a wide variety of substrates, which includes several types of phenolic and nonphenolic hazardous compounds (Agrawal et al. 2018; Jawale et al. 2021). A major reason for the interest in laccase is its broad range of substrate oxidation activity, the absence of cofactors, and the availability of oxygen as an electron acceptor, which makes them a potentially useful and versatile enzyme with number of biotechnological applications. Therefore, laccases are applied in several industrial processes including, pulp and paper (Aracri et al. 2009), textile (Mandic et al. 2019), cosmetics (Shin et al. 2019), pharmaceutical (Yang et al. 2017), food (Singh and Gupta 2020) and in numerous bioremediation processes (Nguyen et al. 2020; Patel and Patel 2020; Souza Vandenberghe et al. 2022).

The multi-copper laccase is broadly occurring in nature, found in bacteria, fungi, plants, archaea, as well as in insects (Uthandi et al. 2010). Among these microbial sources, there is a great deal of research being conducted to find novel microbial species exhibiting unique features to use them for laccase production (Jawale et al. 2021). Moreover, features like small generation time, high proliferation rate, easy to handle and the ability to produce large amount of products using cheap and natural raw materials make them more efficient (Thapa et al. 2020). The bacterial laccase is relatively extremely active and is significantly more stable under high temperatures, high pH conditions, as well as under high chloride concentrations in compare to fungal laccase (Margot et al. 2013). In recent years, significant progress has been made in the screening of bacteria for their laccase production (Akram et al. 2022; Mate and Alcalde 2015). Several studies have highlighted the diversity of bacteria capable of producing laccases. Recent research has expanded our knowledge about laccase-producing bacteria beyond traditional sources. For instance, extremophiles from extreme environments such as hot springs and deep-sea sediments have been identified as potential laccase producers, indicating the broad range of ecological niches for this enzyme (Chandra et al. 2017; Pham et al. 2023; Zhu et al. 2022). By analyzing the genetic material present in environmental samples via metagenomics, researchers have identified previously unknown laccase producers, unlocking new possibilities for industrial applications (Ausec et al. 2011; Dai et al. 2021; Ferrer et al. 2010). Furthermore, recent research efforts have resulted in the discovery of novel laccase variants with unique properties. These enzymes exhibit increased stability, substrate specificity and tolerance to harsh conditions, making them promising candidates for various biotechnological applications (Bisaccia et al. 2023; Chauhan et al. 2017). Automation and high-throughput screening techniques have accelerated the process of identifying laccase-producing bacteria. These methods allow researchers to quickly assess the enzymatic capabilities of a large number of bacterial strains, facilitating the discovery of promising candidates (Rodríguez-Escribano et al. 2017; Sarnaik et al. 2020). Furthermore, several studies reported the production of laccase by bacteria using low-cost agricultural wastes such as sawdust, corn, apple pomace, canola roots, sugarcane bagasse, cotton stalks and wheat bran (Kumar et al. 2022). These agriculture residues pose a serious threat to the environment if not managed properly. Therefore, using these wastes for the degradation and recycling of pollutants has become an important strategy to reduce the disposal problems (Chen et al. 2021).

In the textile and paper industries, thousands of different dyes are produced, released each year, and remain stable under environmental conditions that include chemicals, light exposure, and microbial degradation. In today’s world, over 10,000 synthetic dyes are used (Blánquez et al. 2019). Because of inherent recalcitrance nature of dye, it poses serious health issues. During dye degradation, dye molecules are decolorized and/or detoxified in contaminated environments by polluted effluents. In the past, several physico-chemical technologies have been studied for dye degradation in wastewater, including filtration, adsorption, photodegradation, and irradiation (Parshetti et al. 2006). Conventional physico-chemical treatments are effective, but ineffective when the dye is present in low concentrations, whereas sometimes it also generates toxic compounds (organochlorides and aromatic amines) that could be more toxic than the dye prior to pretreatment (Rueda-Villabona et al. 2021). Pretreatment using bacteria or enzymes has the advantage of total dye decolorization and breakdown of its intermediates as well as decreasing negative environmental effects (Patel and Patel 2020).

Biodegradation of dye is performed enzymatically through reduction, oxidation, hydroxylation and methylation reaction of dye’s structures (Rueda-Villabona et al. 2021). Laccase from bacteria and fungi are reported for their dye’s bioremediation potential. Till date, many dyes like Malachite green, Bromophenol blue, Remazol bright blue R, Methyl orange, Congo red, Coomassie G-250, Crystal violet, Alizarin red, Indigo blue etc., are reported to degrade by microbial laccase (Ardila-Leal et al. 2021). Apart from fungi, bacteria are emerging as a novel source to synthesize laccase with similar potential of fungal enzymes. Because of their various industrial application cost-effective usage, short time enzyme production, broad substrate specificity, bacterial laccase are the potential tools with many advantages (Ben Younes et al. 2021).

Aiming to promote industrial laccase use by isolating efficient laccase producers, we isolated *S. maltophilia* E1 that produces high laccase levels and decolorizes structurally different dyes efficiently. In the present study, isolated *S. maltophilia* E1 produce laccase in the presence of coconut husk as a substrate and decolorized dyes in the absence of a mediator. To optimize laccase production, a response surface methodology (RSM) was used. The decolorized and degraded malachite green was analyzed by gas chromatography–mass spectrometry (GC–MS) analysis. Moreover, the degraded metabolites were further assessed by phytotoxicity test.

## Materials and methods

### Isolation and screening of laccase producing bacteria

The soil sample was collected in sterile tubes from the Amlakhadi located near Ankleshwer, Gujarat Industrial Development Corporation (GIDC) (21°36′55°N, 72°59′52°E), Gujarat, India. The sample was transported immediately to the lab in an icebox for further use. Soil sediment sample was sequentially diluted up to 10^–8^ and then 0.1 mL sample was spread on M162 agar medium supplemented with 2 g/L yeast extract, 2 g/L tryptone, 100 µM CuSO_4_, and 2 mM Guaiacol (Sondhi and Saini 2019). The plates were incubated at 37 ℃ for 48–72 h. The production of laccase was indicated by reddish brown color on the agar plate. Isolates were further screened for their laccase activity, which was carried out by embedding 2,2’azino-bis [3-ethylbenzthiazoline-6-sulphonic acid] (ABTS) and syringaldazine in agar medium separately. Appearances of green color represent the true laccase producer.

### Identification of laccase producing bacterial strain

Identification of potent laccase producing bacterial strains was conducted with the help of 16S rRNA gene sequence analysis. The genomic DNA was isolated through NaCl-cTAB method described by Wilson (2001). The conserved nucleic acid sequences of strains were multiplied by polymerase chain reaction (PCR) utilizing universal primers (reverse-1492r and forward-27f). The amplification system and required conditions were based on those given by Desai and Patel (2019). After sequencing, BLAST-analysis was conducted on the nucleotide sequences obtained using the reference sequences available at NCBI, GenBank.

### Evaluation of laccase production by agro-residues waste

The laccase production study by means of submerge fermentation using commonly available agricultural waste residues i.e., sawdust, coconut husk, rice, wheat, and barley bran as substrate was carried out. The pretreatment for sawdust and coconut husk including, washing with distilled water, 15 min boiling to remove dust particles, and then drying at 60 ℃ in oven. After complete drying, obtained residues were milled through grinder, and passed through a 0.5 mm size sieve for uniform particle size (Prajapati et al. 2021). The laccase production via submerge fermentation was carried by incorporating 1% of each waste residues into 100 mL of M162 broth containing 2 g/L tryptone, 2 g/L yeast extract, and 100 µM CuSO_4_. Precisely, 1% (v/v) active culture (absorbance, A600nm: 0.8) was inoculated in the flask, followed by incubation at 37 ℃ for 48 h. After incubation, flask material was centrifuged at 10,000 rpm for 5 min, and the supernatant was used to measure laccase activity using Guaiacol as a substrate.

### Laccase assay

The enzyme activity was determined through monitoring the oxidation of 1 mL of 10 mM Guaiacol buffer using 3 mL (100 mM) sodium acetate buffer at 470 nm (Margot et al. 2013). Enzyme activity was expressed in International Units (IU), where 1 IU is described as the amount of enzyme essential to oxidize 1 µmol of Guaiacol per min under assay conditions. To calculate laccase activity, the following formula was used (Patel et al. 2009):

$${\text{Enzyme}}\,{\text{activity}}\,\left( {{\text{IU}}/{\text{ml}}} \right)\, = \,\frac{{\left( {{\text{A}}\, \times \,{\text{V}}} \right)}}{{\left( {{\text{t }}\, \times \,{\text{e}}\, \times \,{\text{v}}} \right)}}$$

where, A = Absorbance at 470, V = total volume (mL), t = incubation time (min), e = coefficient of Guaiacol (26.6 m^−1^ M^−1^) at 470 nm, v = enzyme volume (mL).

### Optimization of laccase production: conventional and statistical approach

The optimization of laccase production was initially conducted by conventional one variable at a time (OVAT) method. Parameters including incubation time (0–60 h), initial pH (3.0–9.0), temperature (25–50 ℃), and agitation speed (50–200 rpm) were selected for this study. Under this approach, at a given time, one variable was changed while the rest were kept unchanged. The BBD of the RSM was used for statistical optimization of process parameters. To optimize laccase productions, four functional variables were considered, including (A) coconut husk (g/L), (B) CuSO_4_ (µM), (C) yeast extract (g/L), and (D) pH. The laccase production was taken as dependent response variable (IU/mL). Table 1 shows the upper and lower limits for each variable. Analysis of variance (ANOVA) was used as the statistical method to determine the significance and adequacy of the model. Three-dimensional (3D) graphs, counter plots, and all statistical analyses were performed using Design Expert version 12 (Stat-Ease).

**Table 1:** Variables optimized by BBD for laccase production

Name of variable with code	Unit	Range and levels		
		−1	0	+ 1
Coconut husk (A)	g/L	5	10	15
CuSO_4_ (B)	µM	50	100	150
Yeast extract (C)	g/L	2	4	6
pH (D)		5.5	6	6.5

### Influence of pH and temperature change on laccase activity and stability

The effect of pH variation was examined within the range of 2–10, using glycine–HCl, sodium citrate, carbonate-bicarbonate and potassium phosphate (50 mM) buffer. Temperature effects were examined in the context of 25–60 °C. The investigation revealed that one parameter was different, whereas the other remained constant, and vice-versa. An investigation of the product's stability under pH variations was conducted at 35 °C and pH values between 2 and 10. Using standard assay conditions, residual activity was determined after 6 h. As for thermal stability, the enzyme was incubated at 25, 30, 35, 40, 45, 50, 55 and 60 °C for up to 6 h. Aliquots were collected at regular time interval for testing enzyme activity under standard assay conditions. The Guaiacol was used as a substrate for both experiment, and activity was measured in the form of relative activity (%).

### Dye decolorization and UV- Vis analysis

The optimized crude laccase (culture filtrate) of isolate E1 was further evaluated for its ability to decolorize dyes, such as Acid orange, Acid red 18, Congo red, Crystal violet, Malachite green, Orange B methyl orange, Methyl red, and Methylene blue. For the decolorization experiment, 100–500 ppm concentration dyes were dissolved separately in distilled water and then passed through Whatman No. 1 filter paper. Then, the action of decolorization was commenced by mixing 100 mL of each dye concentration and 10 mL of crude enzyme (51.38 ± 1.11 IU/mL) solution in 0.1 M sodium acetate buffer (pH—4.0) and allowing to stand at 35 ℃ for 24 h. The samples were withdrawn after incubation and the decolorization of dyes using a spectrophotometer at the maximum wavelength of each dye was measured. Controls containing dyes without the enzyme were used as negative controls. Decolorization experiments were conducted in triplicate in all cases. The following formula was used to calculate decolorization:

$${\text{Dye}}\,{\text{decolorization}}\, = \,\frac{{\left( {{\text{Absorbance}}\,{\text{of}}\,{\text{control}}} \right)\,-\,\left( {{\text{Absorbance}}\,{\text{of}}\,{\text{test}}} \right)}}{{\left( {{\text{Absorbance}}\,{\text{of}}\,{\text{control }}} \right)}}\, \times \,100$$

A spectrophotometric analysis of laccase-treated and untreated dye samples was conducted between the wavelengths 300 and 800 nm using a UV–Vis spectrophotometer (Shimadzu, India). For that, dye samples were initially centrifuged at 15,000 rpm for 30 min and then supernatant was extracted with ethyl acetate at an equal volume.

### Extraction of decolorized product of the dyes

Dye degradation assays were used to identify intermediate compounds resulting from dye decolorization. After collecting the decolorized solution, it was mixed thoroughly with an equal volume of ethyl acetate, and the two phases were vortexed. A rotary evaporator was used to collect and dry the separated organic layer (Ghobadi Nejad et al. 2019). The dried residues of test and control sample were further suspended in 3 mL of HPLC-grade methanol, mixed thoroughly, filtered by 0.45 µm filter and prepared for chromatographic analysis.

Gas chromatography mass spectrometry (GC–MS) was used to characterize and identify the degraded products, whereas the untreated (control) solution was used as a reference. The filtered sample was applied for GC–MS with a DB-5 MS column (30 cm × 0.25 mm id., 0.25 mm film thickness) and helium as the carrier gas at a 1 mL/min flow rate. The instrument that was employed for GC–MS analysis was an Agilent (Agilent Technologies, India) 7890 gas chromatograph coupled with a 5975-quadrupole mass spectrometer detector. The injector temperature was allowed to remain at 60 ℃ for 2 min, then 25 ℃/min to 100 ℃ for 0 min and then increased linearly to 250 ℃ for 15 min kept constant. To identify intermediate compounds that result from dye degradation, the NIST Research Library was used to compare spectra of the compounds.

### Phytotoxicity analysis

The toxicity of biodegradable dye metabolites was determined with seeds of *Oryza sativa*. The experiment was carried out using the method proposed by Patel and Patel (2020) with some changes. A total of 20 seeds were surface sterilized using sodium hypochlorite, followed by washing with de-ionized water. Afterwards, seeds were grown in a glass Petri dish lined with 2–3 layers of filter paper that was soaked with a solution, prepared as follows: (1) positive control-distilled water; (2) negative control-untreated dye solution and (3) laccase-treated solution. Petri dish containing seeds were incubated at 37 ℃ temperature. The seeds were regularly watered or irrigated when required to keep the filter paper moist with the respective treatment solution. All the experiments were done in triplicates. Seed germination percentage was calculated by the following formula:

$${\text{Seed}}\,{\text{germination}}\,\left( {\text{\% }} \right)\, = \,\frac{{{\text{Number}}\,{\text{of}}\,{\text{seeds}}\,{\text{germinated}}}}{{{\text{Total}}\,{\text{number}}\,{\text{of}}\,{\text{seeds}}}}\, \times \,100$$

After 7 days of different treatment, seedling length (total plumule + radicle lengths) was measured using scale in centimeters. Similarly, vigor index (unit less) calculated by the following formula:

$${\text{Vigor}}\,{\text{index}}\, = \,{\text{seedling}}\,{\text{length}}\, \times \,{\text{seed}}\,{\text{germination}}\,{\text{percentage}}$$

### Statistical analysis

Experiments such as screening, decolorization assays, optimization studies, enzymes characterization, and toxicity tests were carried out in triplicate. To analyze the data from the toxicity assay, a one-way ANOVA and a Tukey's post hoc test were used to perform a statistical analysis. Whereas, OVAT optimization and laccase production using low-cost substrates were analyzed statistically using one-way ANOVA and Dunnett’s multiple comparison as post hoc test. Graph Pad Prism 5.0 software was used for the statistical analysis, and the significance level was set at *p* < 0.05.

## Results

### Isolation and identification of potent laccase producer

**Fig. 1:**
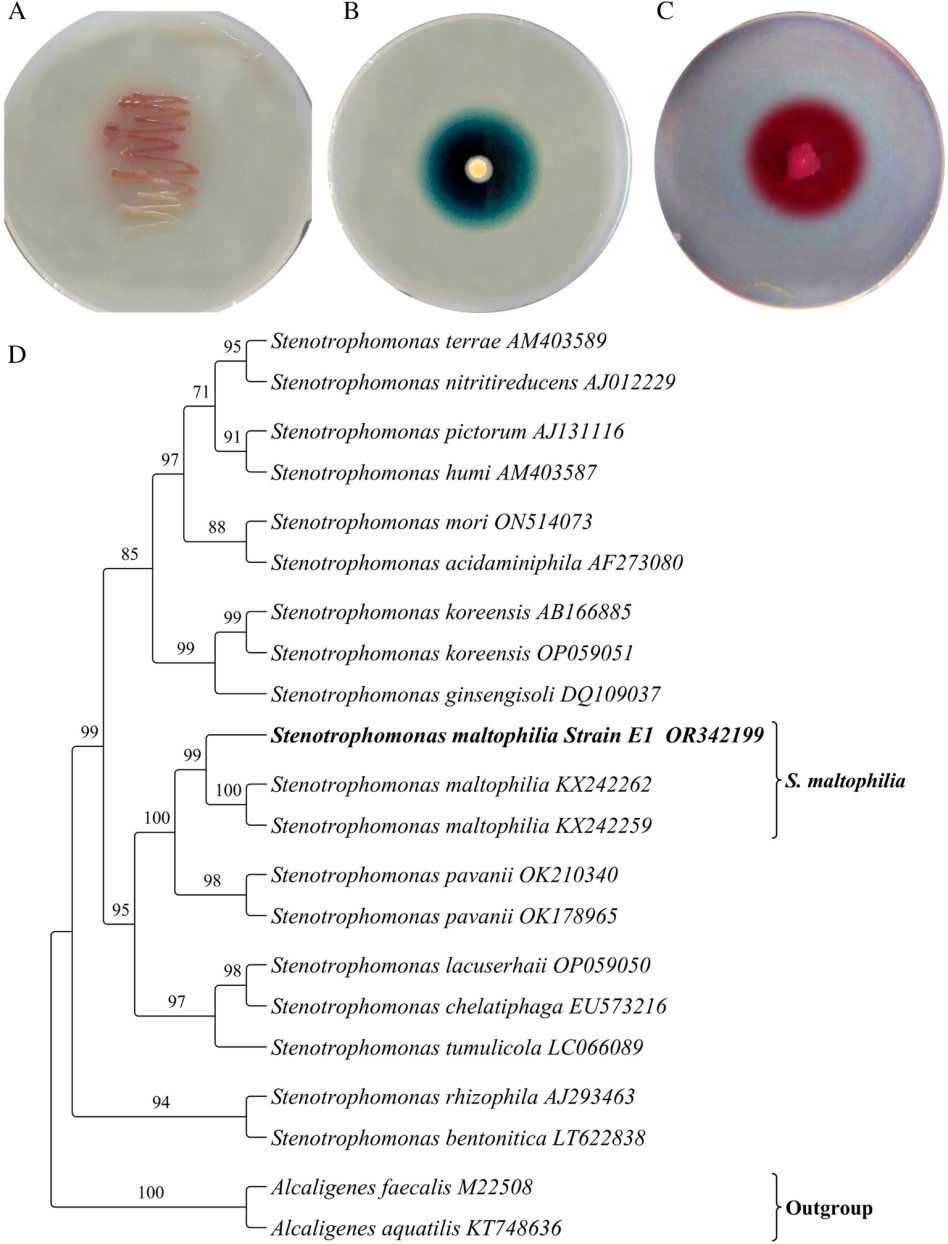
Screening of isolate E1 form laccase production on **A** Guaicol **B** ABTS, and **C** syringaldazine embedded in agar plates **D** phylogenetic analysis

Based on morphological characters, a total of 3 bacterial strains showed laccase production on guaicol agar, whereas only one isolate E1 exhibited ATBS and Syringaldazine substrate oxidation capacity (Fig. 1A–C). The potent true laccase producer isolate E1 was further identified via nucleotide gene sequencing method. The partial amplified 16S rRNA was performed and the resulting sequence (1400 bp) was identified as *Stenotrophomonas maltophilia* E1. The nucleotide sequence have been submitted to NCBI GenBank with accession number OR342199. The BLAST analysis of the 16S rRNA sequence comparison showed that strain E1 had the most closely related phylogenetic linkage to the genus *Stenotrophomonas*. The results of phylogenetic analysis indicated that the strain *S. maltophilia* E1 is closely associated with other strains of *Stenotrophomonas* (Fig. 1D).

### Evaluation of various low-cost substrates for laccase production

**Fig. 2:**
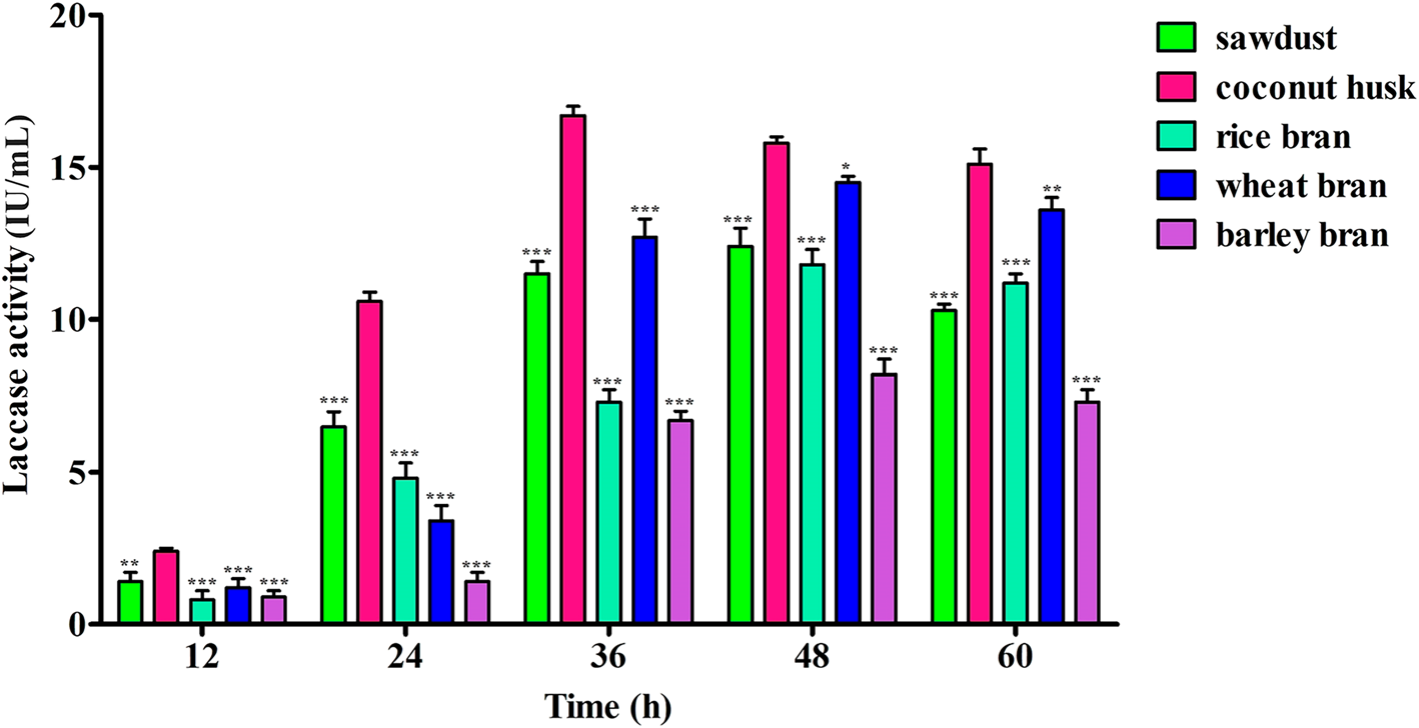
Evaluation of laccase production using agricultural waste residue. Statistical significance; ns > 0.05, *p < 0.05, **p < 0.005, ***p < 0.0005

Several easily available and cheap agriculture waste residues like sawdust, coconut husk, rice, wheat and barley bran were comparatively examined for production of laccase (Fig. 2). The laccase production was checked at various time interval under the shaking condition. Production of laccase was found higher during the incubation period of 24–48 h. The highest laccase production was noted in the presence of coconut husk (16.7 ± 0.3 IU/mL), followed by wheat bran (14.5 ± 0.2 IU/mL), sawdust (12.4 ± 0.6 IU/mL), rice bran (11.8 ± 0.5 IU/mL), and barley bran (8.2 ± 0.5 IU/mL).

### Optimization of laccase production

**Fig. 3:**
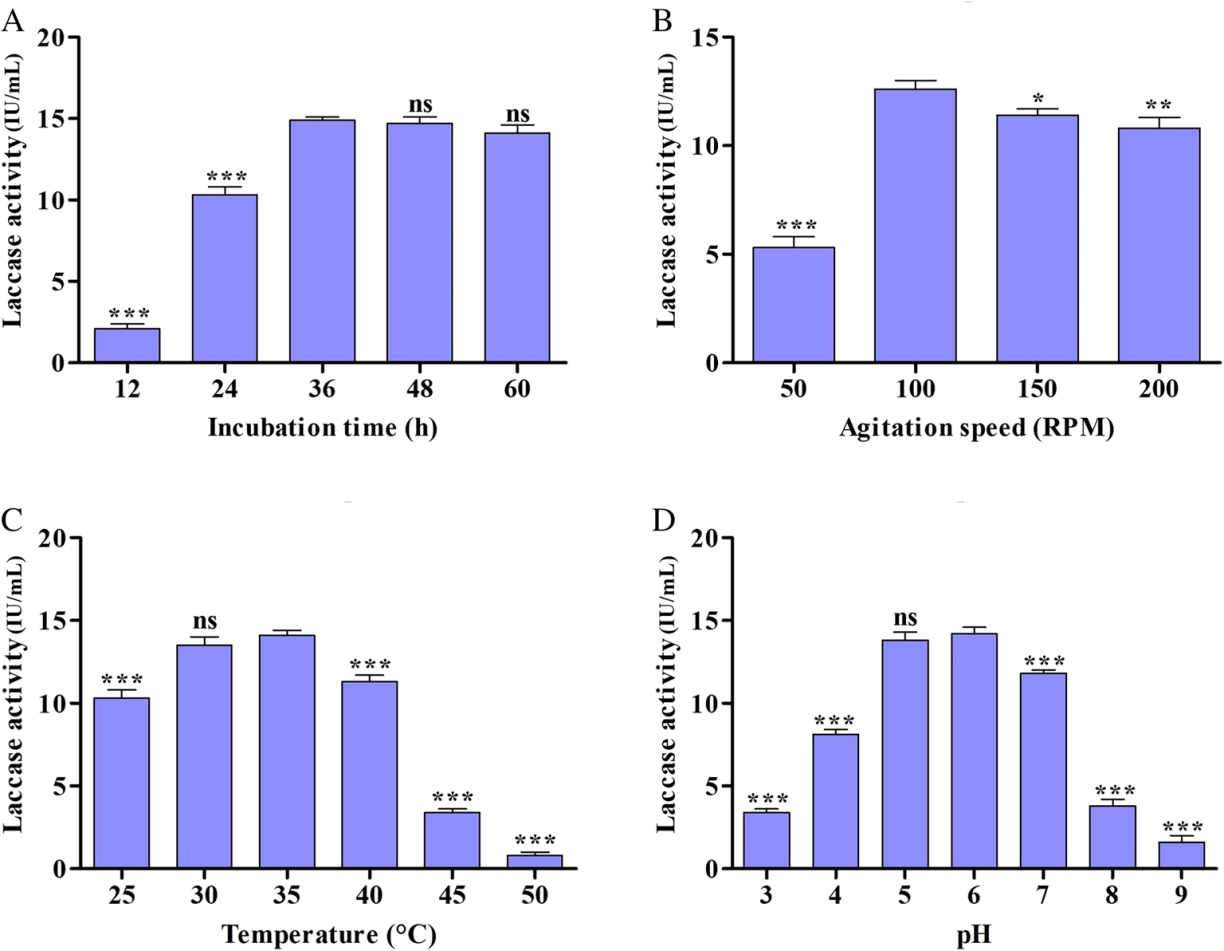
Optimization of fermentation parameters by OVAT approach for maximum laccase production. **a** effect of incubation time, **b** effect of agitation, **c** effect of temperature, and **d** effect of pH on enzyme production. Statistical significance; ns > 0.05, *p < 0.05, **p < 0.005, ***p < 0.0005

The effect of different factors on the laccase production by *S. maltophilia* E1 was initially identified through the OVAT method followed by the potential variables study via BBD approach. Result of the OVAT approach displayed that the maximum laccase production (14.9 ± 0.2 IU/mL) was achieved at 36 h incubation time (Fig. 3A), whereas 100 rpm was best suited for maximum laccase (12.6 ± 0.4 IU/mL) production (Fig. 3B). As presented in Fig. 3C, maximum laccase production achieved at 35 ℃ temperature (14.1 ± 0.3 IU/mL) and pH 6 (14.2 ± 0.4 IU/mL) (Fig. 3D).

In this study, the selected parameters such as concentrations of coconut husk, CuSO_4_, yeast extract, and pH were studied to determine their effect on the laccase production via BBD experiment. The design matrix used for the experiment and summary of the results are presented in Table 2. A total of 29 experimental runs were conducted in flask containing production medium that incubated for 42 h under shaking conditions. The strain E1 exhibited a significant difference (maximum—51.38 ± 1.11 IU/mL; minimum—13.5 IU/mL) in laccase production. Table 2 shows the apparent influence of process variables with respect to their range. To determine the effect of each independent factor, multiple regression analysis was performed, and the following polynomial equation was derived from the regression analysis:

$${\text{Y}}\, = \, + \,51.38\, - \,4.43\,{\text{A}}\, - \,0.7333\,{\text{B}}\, - \,11.14\,{\text{C}}\, - \,4.13\,{\text{D}}\, - \,7.35\,{\text{AB}}\, + \,0.225\,{\text{AC}}\, - \,4.3\,{\text{AD}}\, - \,2.35\,{\text{BC}}\, - \,1.6\,{\text{BD}}\, + \,0.35\,{\text{CD}}\, - \,11.48\,{\text{A}}^2 \, - \,9.87\,{\text{B}}^2 \, - \,9.61\,{\text{C}}^2 \, - \,7.19\,{\text{D}}^2$$

**Table 2:** Box Behnken design along with experimental and predicted values of dependent variable for laccase production

Run	A: coconut husk (g/L)	B: CuSO_4_ (µM)	C: yeast extract (g/L)	D: pH	Laccase production (IU/mL)	
					Experimental	Predicted
1	−1	0	1	0	21.8	23.35
2	−1	0	−1	0	46.7	46.08
3	0	−1	0	−1	38.1	37.58
4	0	−1	1	0	24.4	23.85
5	1	0	1	0	13.5	14.95
6	0	1	0	1	26.5	27.85
7	0	0	0	0	52.4	51.38
8	1	0	0	−1	38.7	36.71
9	0	0	0	0	51.7	51.38
10	1	1	0	0	17.4	17.52
11	−1	0	0	−1	36.5	36.96
12	0	0	0	0	50.3	51.38
13	0	0	0	0	50.1	51.38
14	0	0	−1	−1	49.2	50.2
15	0	−1	−1	0	40.8	41.43
16	0	1	0	−1	38.3	39.32
17	0	1	−1	0	44.1	44.66
18	0	−1	0	1	32.7	32.52
19	−1	−1	0	0	28.8	27.84
20	1	−1	0	0	32.1	33.69
21	0	0	1	1	21.5	19.65
22	1	0	0	1	20.3	19.85
23	0	0	0	0	52.4	51.38
24	0	1	1	0	18.3	17.68
25	−1	1	0	0	43.5	41.07
26	0	0	1	−1	27.2	27.22
27	−1	0	0	1	35.3	37.3
28	0	0	−1	1	42.1	41.24
29	1	0	−1	0	37.5	36.78

where, Y represents laccase production (IU/mL); A represents concentration of coconut husk; B represents concentration of CuSO_4_; C represents concentration of yeast extract; and D is the initial pH.

ANOVA analysis was used to determine if the model is suitable for estimating the impact of the numerous variables. This was then tested using Fisher's statistical analysis, which led to the results presented in Table 3. A correlation coefficient of *R*^2^ = 0.9902 was found, indicating that the model covered roughly 99.02% of responses, indicating that the experimental and predicted values were related. According to the model *F*-value and p-value, the model is significant with a *F*-value of 101.52 and a *p*-value of < 0.0001. In this case, the *F*-value of the data was calculated with a 'Lack of Fit' of 2.63, which indicates that the 'Lack of Fit' is also not statistically significant when compared with the pure error. The coefficient of variation (CV) indicates the degree of precision, and it is low (4.62), which means the experiment performed is highly reliable. In this case, a very low probability score (*P* < 0.05) indicate that model parameters were significant in predicting the response variable.

**Table 3:** ANOVA for laccase production as a function of independent variables

Source	Sum of squares	*df*	Mean Square	*F* value	*p* value Probe > *F*
Model	3839.97	14	274.28	101.52	< 0.0001*
A-coconut husk	234.97	1	234.97	86.97	< 0.0001*
B-CuSO_4_	6.45	1	6.45	2.39	0.1445
C-yeast extract	1489.64	1	1489.64	551.37	< 0.0001*
D-pH	205.01	1	205.01	75.88	< 0.0001*
AB	216.09	1	216.09	79.98	< 0.0001*
AC	0.2025	1	0.2025	0.075	0.7883
AD	73.96	1	73.96	27.38	0.0001*
BC	22.09	1	22.09	8.18	0.0126*
BD	10.24	1	10.24	3.79	0.0719
CD	0.49	1	0.49	0.1814	0.6767
A^2^	855.1	1	855.1	316.51	< 0.0001*
B^2^	631.79	1	631.79	233.85	< 0.0001*
C^2^	598.63	1	598.63	221.57	< 0.0001*
D^2^	335.71	1	335.71	124.26	< 0.0001*
Residual	37.82	14	2.7		
Lack of fit	32.84	10	3.28	2.63	0.1818^ns^
Pure error	4.99	4	1.25		
Cor Total	3877.8	28			

*R*^2^ = 0.9902; Adj-*R*^2^ = 0.9805

*df* degree of freedom, *ns* not significant

^*^significant

**Fig. 4:**
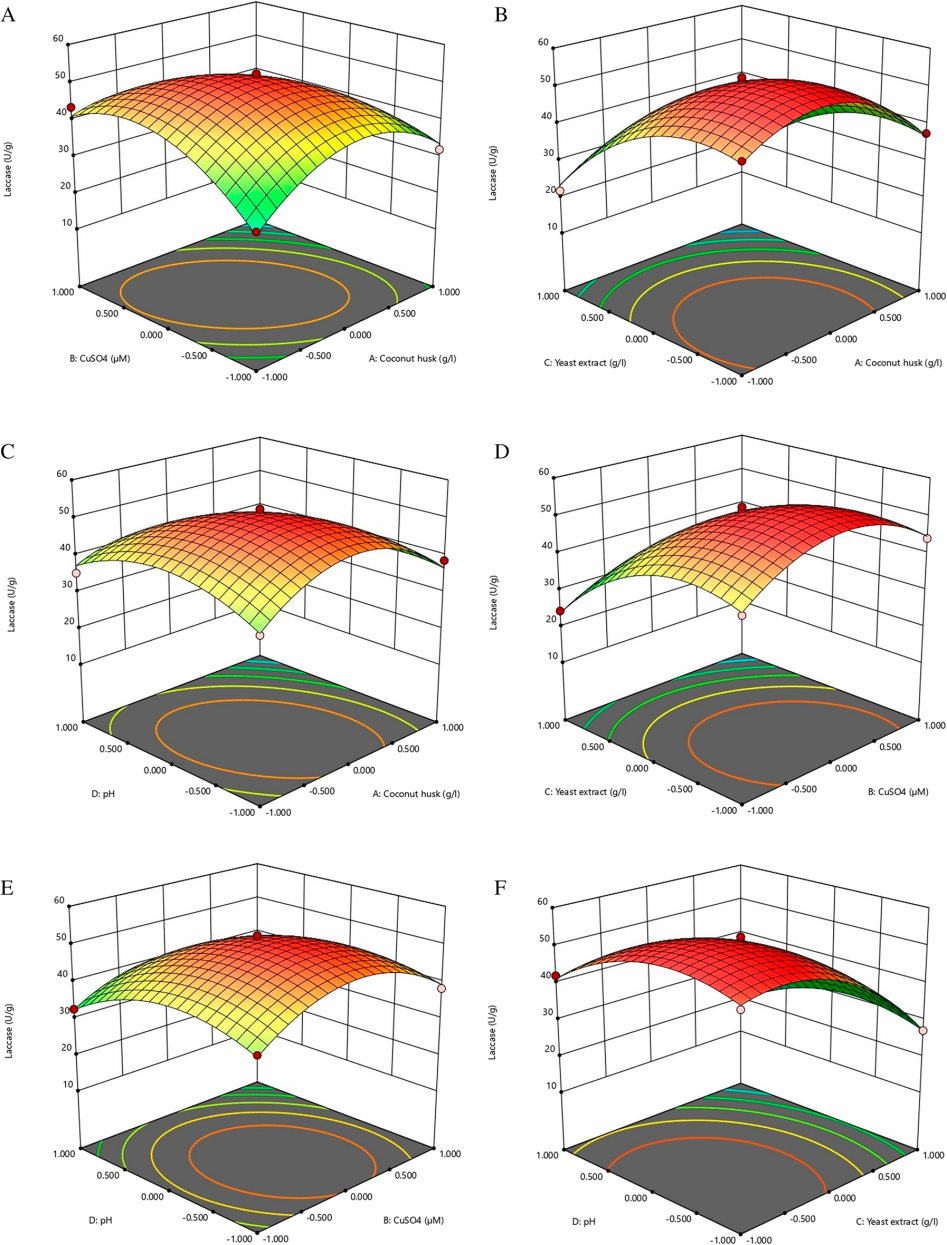
3D surface plots showing the effect of **a** coconut husk and CuSO_4_; **b** coconut husk and yeast extract; **c** coconut husk and initial pH; **d** CuSO_4_ and yeast extract; **e** CuSO_4_ and initial pH; **f** yeast extract and initial pH on laccase production

To better understand the results of the study, a 3D response surface plot is presented in Fig. 4, where it is clear that the theoretical maximum laccase production for each graph is relatively similar to the other graphs. The slope of the response surface provides insight into the interaction of various factors and the relative importance of laccase production. The central point in the counterplots represents the greatest potential for laccase production. Thus, the optimum medium conditions for laccase production were concluded to be 10 mg/L coconut husk concentration, 100 µM CuSO_4_ concentration, 4.0 mg/L yeast extract concentration, and 6.0 initial pH. In the optimized production medium, the production of laccase reached 51.38 ± 1.11 IU/mL, which is basically consistent with the model predicted theoretical value (51.38 IU/mL).

### Effect of pH and temperature

**Fig. 5:**
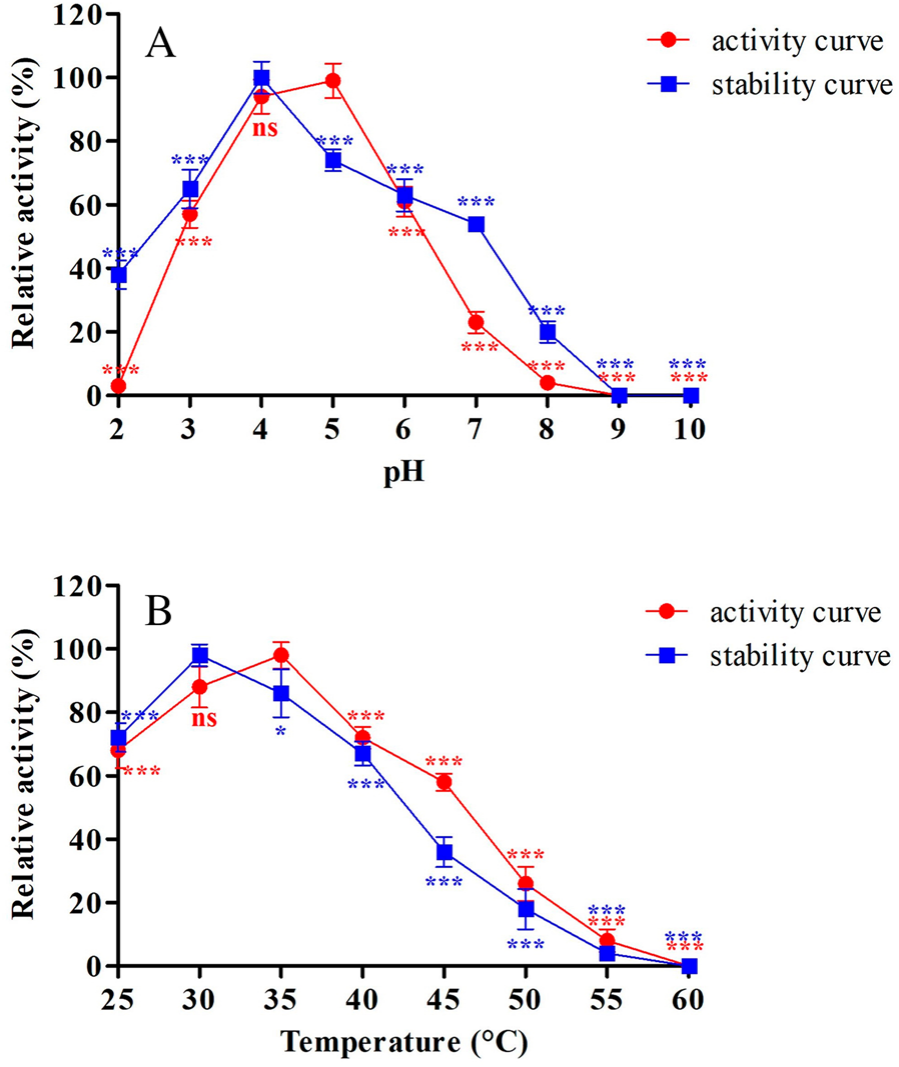
Effect of **a** pH and **b** temperature on laccase activity and stability. Statistical significance; ns > 0.05, *p < 0.05, **p < 0.005, ***p < 0.0005

The obtained results revealed the optimal pH value of the crude laccase was 5 towards ABTS, with roughly 40% activity loss at pH 6.0 and 96% activity loss at pH 8.0. Interestingly, the oxidizing activity almost vanished, when the assay pH was raised above 9.0 (Fig. 5A). A temperature of 35 °C was found to be the optimal temperature for crude laccase. A total of about 42% of the total activity was retained, when it was assayed at 45 °C, whereas the oxidizing activity almost completely disappeared, when the assay temperature was raised above 60 °C (Fig. 5B). In case of stability, there was a strong stability in the pH range of 3.0–6.0 during the assay. Furthermore, 60% of the total activity was still present after 6 h of incubation (Fig. 5A). When crude laccase was grown at pH 4.0, the oxidizing activity of crude laccase exhibited superior stability, followed by a constant decline over time. According to the thermal stability test, crude laccase was very stable at temperatures between 25 and 40 °C for 6 h (Fig. 5B). When incubated at 30 ℃, crude laccase showed highest stability. However, it showed a slight decline of oxidizing activity at 35 ℃ with the 56% residual activity.

### Dye decolorization and degradation study by crude laccase

#### Decolorization study by UV–Vis Spectrophotometry

**Fig. 6:**
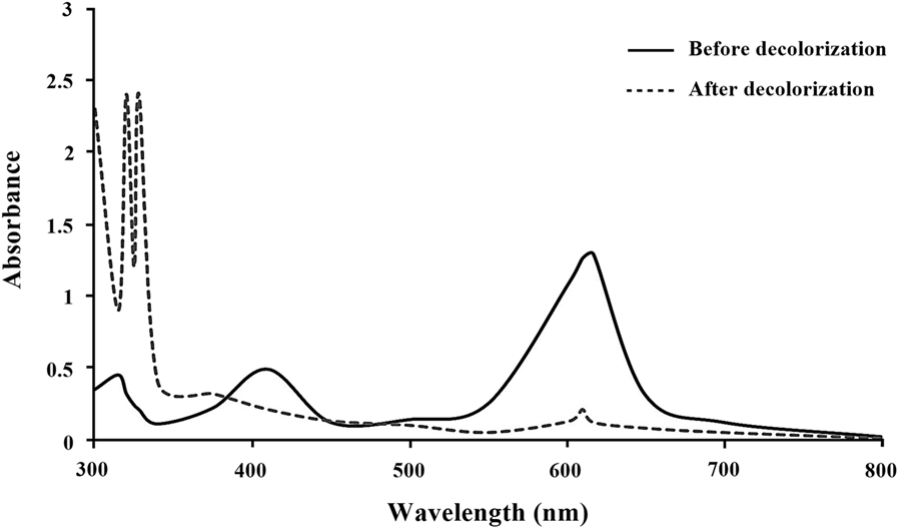
UV–Vis spectrometry analysis of malachite green containing solution before and after treatment with laccase

The initial exploration of crude laccase for nine different dyes decolorization was investigated using UV–Vis Spectrophotometry (Table 4). The results shown that crude laccase completely decolorized Malachite green up-to 300 ppm concentration. Whereas, Methyl red, Congo red, and Methyl orange were decolorized 95.4 ± 0.8, 92.6 ± 1.2, and 90.4 ± 1.2 at 100 ppm concentration, respectively. Further, the UV- Vis spectral analysis of untreated Malachite green shown major absorbance peak at 416 nm, which was completely disappear and giving complete decolorization of dyes after 24 h laccase treatment. However, three minor new peaks appeared at 320, 328, and 610 nm (Fig. 6), which indicates the generation of new intermediate metabolites after laccase treatment.

**Table 4:** Analyzing dye decolorization and seed germination study

Dye name	Decolorization (%)					Germination (%)
	100 ppm	200 ppm	300 ppm	400 ppm	500 ppm	
Acid orange	87.6 ± 1.7	85.3 ± 2.6	80.3 ± 1.1	75.4 ± 3.3	66.7 ± 3.1	65.33 ± 4.72
Acid red 18	68.3 ± 2.8	67.8 ± 3.1	65.9 ± 1.6	63.1 ± 2.5	62.2 ± 2.5	48.74 ± 3.21
Congo red	92.6 ± 1.2	91.9 ± 0.4	88.6 ± 1.5	82.4 ± 1.6	75.9 ± 3.6	16.15 ± 2.15
Crystal violet	21.3 ± 0.8	20.4 ± 2.3	15.4 ± 3.5	13.7 ± 0.8	12.1 ± 1.3	62.4 ± 4.33
Malachite green	99.4 ± 0.2	98.8 ± 0.6	98.3 ± 0.8	94.1 ± 2.2	91.8 ± 2.0	92.1 ± 3.5
Methyl orange	90.4 ± 1.2	87.8 ± 3.3	82.6 ± 0.5	71.3 ± 0.8	62.3 ± 5.1	28.63 ± 2.1
Methyl red	95.4 ± 0.8	91 ± 1.0	84.6 ± 1.6	72.4 ± 1.1	68.1 ± 2.6	34.15 ± 1.63
Methylene blue	33.9 ± 1.6	32.4 ± 3.9	32.1 ± 0.9	25.7 ± 2.7	22.8 ± 1.5	85.3 ± 2.6
Orange B	54.3 ± 1.5	51.4 ± 1.5	49.8 ± 1.1	47.3 ± 1.9	46.6 ± 1.8	12.4 ± 2.2

Germination (%) of *Oryza sativa* seeds was determined for all dyes taken in experiment at 100 ppm concentration after 24 h laccase treatment

#### Identification of degraded product analysis by GC–MS

**Fig. 7:**
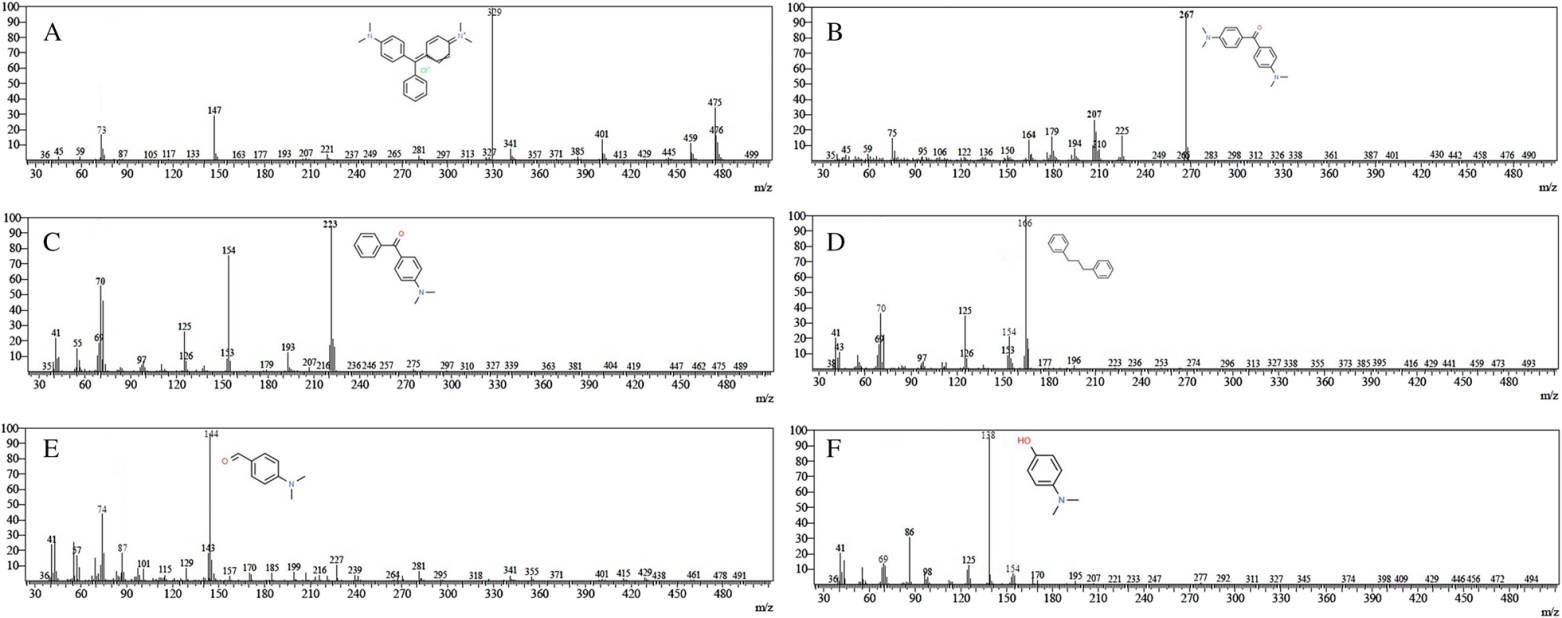
The mass spectra of the degradation products of malachite green by laccase. **A** malachite green (329 m*/z*), **B** Michler’s ketone Bis(4-(dimethylamino) phenyl) methanone (267 m*/z*), **C** 4-(dimethylamino) benzophenone (223 m*/z*), **D** dibenzylmethane (166 m*/z*), **E** 4-(dimethylamino) benzaldehyde (145 m*/z*), **F** 4-(dimethylamino) phenol (138 m*/z*)

The metabolites formed during Malachite green breakdown were studied by GC–MS. They were identified by comparing the resemblance of their fragment RTs and molecular ions that were similar to those of authentic compounds in the database of NIST library. As shown in Fig. 7A for the mass of untreated Malachite green (329 m*/z*), the peak disappeared after enzymatic treatment and new peaks were generated (Fig. 7B–F). The intermediates include Michler’s ketone Bis(4-(dimethylamino) phenyl) methanone (267 m*/z*), 4-(dimethylamino) benzophenone (223 m*/z*), dibenzylmethane (166 m*/z*), 4-(dimethylamino) benzaldehyde (145 m*/z*), and 4-(dimethylamino) phenol (138 m*/z*) were identified (Fig. 7).

### Phytotoxicity assay

The toxicity of Malachite green and its laccase mediated decolorization products were performed with *O. sativa* seeds. The germination index was used as an indicator of phytotoxicity in Petri dishes. The relative sensitivity of *O. sativa* seeds toward laccase treated and untreated Malachite green solutions are summarized in Table 5. Whereas, germination index of all laccase treated at 100 ppm dye is represented in Table 4. The results of the phytotoxicity study showed about 42.15% inhibition of the germination of *O. sativa* seeds soaked in the laccase untreated Malachite green. Whereas, laccase-treated Malachite green showed only 4.52% inhibition of the germination of *O. sativa* seeds compared to control. The seedling lengths of seeds germinated in the untreated dye solution were found to be almost fifteen times lower than those of the seeds germinated in the degradation metabolites (*P* < 0.05). Whereas, a significant improvement in the seedling length was observed in the extracted decolorization products (Table 4). The vigor index of laccase treated dye solution was comprehensively higher than untreated dye solutions (Table 4).

**Table 5:** Phytotoxicity assessment of malachite green metabolites with laccase treatment by *Oryza sativa* assay

Analysis items	Control	Untreated	Treated
Germination (%)	95.66 ± 1.52	55.33 ± 3.39^***^	91.33 ± 4.72^ns^
Seedling length (cm)	4.93 ± 0.63	0.6 ± 0.2^***^	4.16 ± 0.28^ns^
Vigor index	471.83 ± 59.96	33.06 ± 10.31^***^	381.63 ± 50.94^ns^

Statistical significance; ns > 0.05, *p < 0.05, **p < 0.005, ***p < 0.0005

## Discussion

Amlakhadi (India), which has been exposed to a variety of xenobiotics released by industrial effluents for a long period of time. At this type of anthropogenic site, the microbial community plays a significant role in biodetoxification and biotransformation of many toxic compounds (Pathak et al. 2009). Due to the prolong exposure of organic dyes, solvent and other compounds, microorganisms have evolved and adapted which can utilize and detoxify these compounds to survive (Patel et al. 2012). Organic effluents from the dye industries have shown negative impact on the environment, because they are often discharged into water bodies, which are used in irrigation for agricultural purposes (Leo et al. 2019). Moreover, effluents generated from the dye and textile industries contain a wide variety of phenolic, as well as non-phenolic substances that contribute to the growth of laccase producing microbes (Deepa et al. 2020). The role of laccase in various applications including dye decolorizing and detoxification are already established by many researchers (Iark et al. 2019; Leo et al. 2019). Therefore, the main objective of the present study was to isolate the bacterial strains having laccase producing ability, and then applied it for various dye decolorization experiment. For that, initially the bacterial strains were screened on Guaicol agar, and then positive isolates were further screened on ATBS and Syringaldazine substrate to determine the true laccase production ability. The isolate named E1 was successfully passed the screening, and it was identified as *Stenotrophomonas maltophilia* E1 by 16S rRNA sequencing techniques. Most commonly, white rot fungi produce laccase, but there are also bacteria that can produce laccase (Ben Younes et al. 2021). We believe that the present study is one of the few reports that deal with the screening of bacterial laccases as far as our knowledge. As a result of a literature survey, it was shown that some *S. maltophilia* E1 strains had been implicated in the production of laccase (Galai et al. 2009; Alaya et al. 2021).

Laccase is an enzyme that can oxidize a wide variety of substrates. This makes it an interesting subject for research on its characteristics and potential uses in industrial processes (Zhu et al. 2016). A robust biotechnological approach for producing extracellular laccase is to induce the production of the enzyme by microbes using agro-residues that are cheap and readily available. In the present study, sawdust, coconut husk, rice, wheat and barley bran were selected for the laccase production in which coconut husk was appeared as best substrate. The literature study shows that wheat bran, wheat straw, rice bran, sawdust, corn, apple pomace, canola roots, sugarcane bagasse, cotton stalk,s etc., were identified as potential agro-residue for laccase production through fungi (Ghosh and Ghosh 2017; Wang et al. 2019). There is a maximum production of laccase by *Bacillus aquimaris* AKRCo2 (4.58 U/mL), when rice bran was used as a substrate for fermentation (Kumar et al. 2022). A comparison of the laccase production of *S. maltophilia* E1 and other reported bacterial strains using a cheap source of substrate is presented in Table 6.

**Table 6:** Production of laccase by *S. maltophilia* E1 (present study) and other bacterial strains from various sources and conditions using low-cost substrates

Bacterial strain	Substrates	Conditions	Activity (IU/mL)	References
*S. maltophilia* E1	Sawdust	Incubation time—24–48 h; temp—37 ℃	12.4	Present study
	Coconut husk		16.7	
	Rice bran		11.8	
	Wheat bran		14.5	
	Barley bran		8.2	
*Bacillus aquimaris* AKRC02	Potato peel	Incubation time—144 h; temp—37 ℃; pH −7.0	1.24	Kumar et al. (2022)
	Banana peel		0.94	
	Sawdust		2.07	
	Pea peel		0.85	
	Wheat bran		3.74	
	Orange peel		0.96	
	Rice bran		4.58	
*Pseudomonas* sp. S2	Potato peel	Incubation time—48 h; temp—37 ℃	1.87	Chauhan and Jha (2018)
	Saw dust		1.42	
*Bacillus subtilis* MTCC 2414	Rice bran	Incubation time—24 h	134.8	Muthukumarasamy et al. (2015)
	Wheat bran		117.6	
	Sawdust		71.2	
	Banana peel		75.6	
*Stenotrophomonas maltophila* BIJ16	Maize stover	Incubation time—96 h; temp—30 ℃; pH −5.0; RPM—140	28.68	Unuofin et al. (2019)
*Citrobacter freundii* LLJ16			27.28	

There are number of factors that affect the metabolic activity of bacterial cells for laccase production, such as the pH of the initial medium, temperature, and the agitation speed of the medium (Kumar et al. 2022). The various culture medium pH affects the laccase productivity. The bacterial isolate E1 shown maximum laccase production in acidic pH (6.0) condition. It was found that a further rise in pH did not result in an increase in the production of laccase. It can be attributed to the poor growth of the organism at an elevated pH, which may be affecting the production of laccase as a result (Patel and Gupte 2016). Temperature is another significant factor that affects the fermentation process due to bacterial growth and enzyme production being sensitive to temperature. Isolate E1 showed maximum laccase production at 35 ℃ temperature. There is a direct link between higher temperatures and adverse effects on the metabolic activity of microbes, thus causing to the denaturation of enzymes (Desai and Patel 2019; Patel and Dudhagara 2020). However, lower temperature (< 20 ℃) did not support bacterial growth, thus leading to reduced enzyme production (Deepa et al. 2020). Earlier studies suggested that the lower the temperature, the slower the metabolic rate of the bacteria, as well as fungi, which led to the reduction in laccase production. However, some researchers have reported an optimal mesophilic temperature range for laccase production using bacteria and fungi (Patel and Gupte 2016; Wang et al. 2019). As a result, isolates exhibiting optimum laccase production at mesophilic temperatures are always preferred for industrial laccase production (Contesini et al. 2018). Furthermore, agitation speeds affect microbial growth by improving dissolved oxygen in the culture medium, which directly affects enzyme production (Patel and Bhaskaran 2020). A maximum laccase production by isolate E1 was achieved after 24 h of incubation, with a slight decrease on subsequent incubations. The reduction in enzyme production during prolonged incubation might be attributed to the inhibition effect of metabolites secreted by the microbes themselves within the medium during incubation (Mehandia et al. 2020).

The RSM approach was applied for the laccase production which was carried out using four independent variables i.e., coconut husk, CuSO_4_, yeast extract and initial pH. There was a non-significant value of lack of fit found in the statistical analyses (*p* > 0.05), and a highly significant level of performance of the model measured at the parameter level (*p* < 0.0001), which indicates that the mode is capable of being accurately predicted by the variation of the parameters (Prajapati et al. 2021). Moreover, the generated model fit goes very well with the experimental results, which indicates the model was significant. The model presented values for the predicted responses that were very close to the actual values of the experimental responses (Table 2), which is consistent with the result of *R*^2^ and *R*^2^ –adj (Table 3). The response surface 3D graph plotted from equation (mention above) illustrates the influence of variables along with their interactions (Fig. 4). However, the yield of the laccase production initially increased with lower concentration up-to optimal value, and then after it declines gradually with higher concentration of variables. The substrate coconut husk having high lignin content and first time laccase production capability of white-rot fungus *Pycnoporus sanguineus* using coconut husk was evaluated by Karim and Annuar (2009). In present study, 10 mg/L coconut husk concentration was observed optimum for laccase production. The CuSO_4_ was characterized as an inducer for laccase production. According to the Buddhika et al. (2021), in addition to being an essential component of cell growth and laccase gene transcription, copper and its metabolism also play an important role in laccase activity. The nitrogen source yeast extracts support bacterial growth and induce laccase production. Zhu et al. (2016) observed and reported that copper and yeast extract were the most powerful inducers of laccase production in *Pleurotus ostreatus* (ATCC 52857). pH of the medium plays an important role for the cell growth and laccase production. Generally, acidic pHs are favorable for laccase production for fungi, whereas for bacteria it is generally natural (Chauhan et al. 2017). Our findings showed that isolate E1 produce optimum laccase under acidic condition (pH 6.0). Kuddus et al. (2013) showed higher laccase production at pH 7.0 in *P. putida.* The optimization study helps to determine exact concentration for maximum product formation which makes process economical.

Enzyme characterization is an important criteria for their application in various fields. Currently fungal laccase is used in majority of biotechnological application. It has been found that the fungal enzymes are able to function efficiently only under slightly acidic conditions (5 > and < 7), while for catalytic activity to occur, a temperature range of 20–35 °C has been found to be most suitable (Abdelgalil et al. 2020). On the other hand, very little is known about bacterial laccase, which has wide substrate specificities that can be utilized in industrial settings (Chandra and Chowdhary 2015). In this study, laccase shown optimum activity at pH—4.0, while remaining stable at pH—5.0. This result is consistent with that attained by Abdelgalil et al. (2020). They tested purified laccase, isolated from *Alcaligenes faecalis* demonstrating maximum activity at pH—4.0. In contrast to this study, laccase from *Anoxybacillus aydernsis* showed optimum activity at pH—7.0, and most stable at pH—7.5. The optimum temperature for crude laccase of isolate E1 was 35 ℃, whereas showing higher stability at wide temperature range. The findings of the present study are similar with several previous studies (Singh et al. 2007; Bozoglu et al. 2013).

**Fig. 8:**
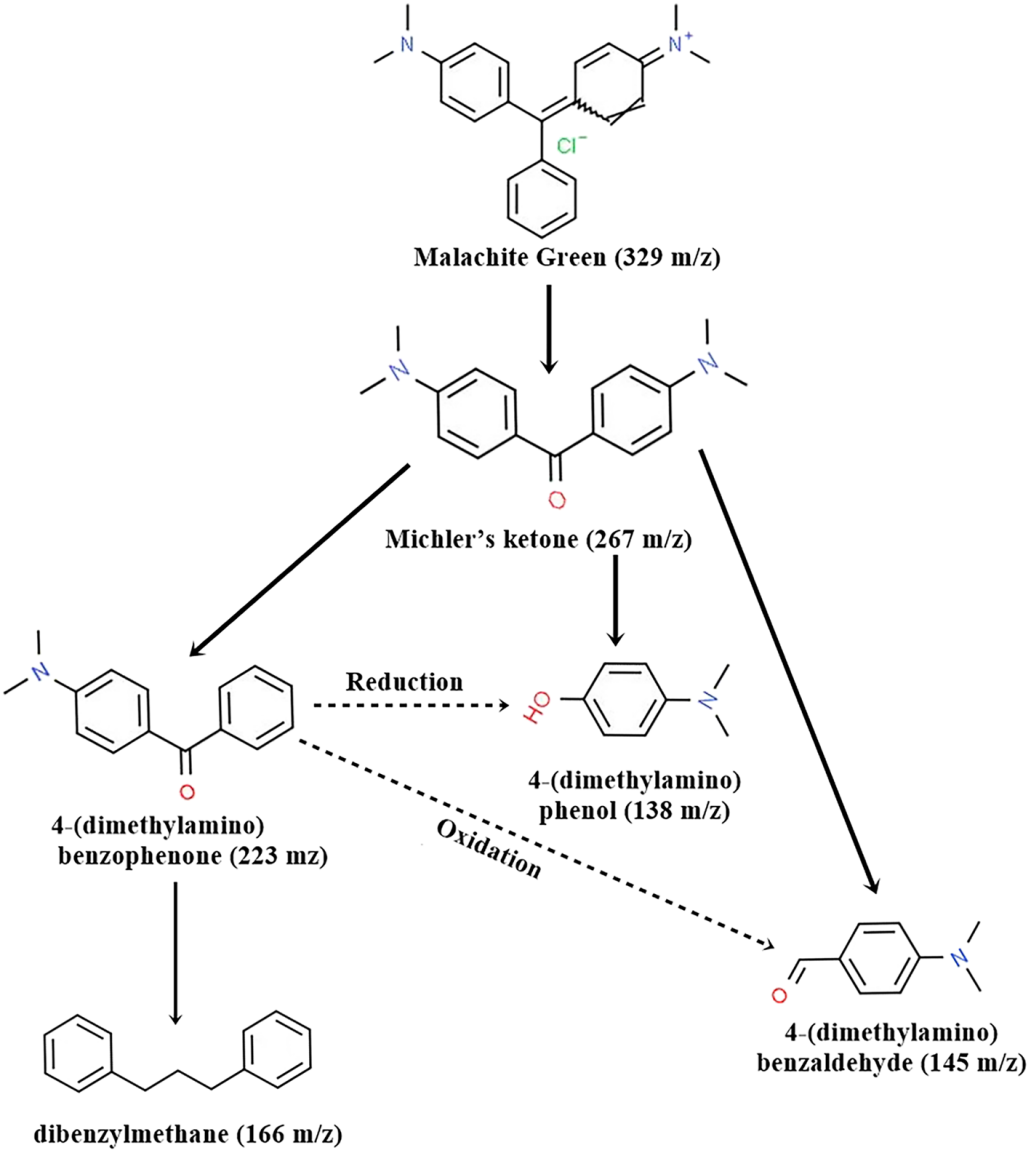
A proposed biodegradation pathways inferred from GC–MS analysis of the products generated during the degradation of malachite green by laccase isolated from *S. maltophilia* E1

Additionally, the laccase form the isolate E1 showed decolorization and degradation ability towards different varieties of synthetic dyes that is same as most studied bacterial laccase (Khan et al. 2013; Lončar et al. 2014; Feng et al. 2015; Abdelgalil et al. 2020). Except Crystal violet and Methylene blue, other seven dyes decolorized more than 80% at 100 ppm concentration. Apart from this, Malachite green decolorized 91.8 ± 2.0% at 500 ppm concentration. Therefore, we can conclude that strain E1 laccase can be considered as a potent decolorizing enzyme. The bacterial laccase is capable of oxidizing numerous benzene ring containing compounds, and it plays a crucial role in textile effluent detoxification (Chandra and Chowdhary 2015). It is well established that the gene ‘lac’ codes for the laccase, which is known as the Malachite green degrader (Yang et al. 2015; Alaya et al. 2021). The Michler’s ketone Bis(4-(dimethylamino) phenyl) methanone, 4-(dimethylamino) benzophenone, dibenzylmethane, 4-(dimethylamino) benzaldehyde, and 4-(dimethylamino) phenol were found after malachite green degradation by laccase treatment. Therefore, a possible proposed degradation pathway for the degradation of malachite green by laccase of *S. maltophilia* E1 is presented in Fig. 8. The result of present study is consistent with previous studies, which also detected these types of metabolites after treating the samples with laccase (Yang et al. 2015; Ghobadi Nejad et al. 2019) and with bacterial degradation (Chen et al. 2009; Du et al. 2011) of Malachite green. A comparison of the degradation of Malachite green by laccase of *S. maltophilia* E1 and other reported microbial strains is presented in Table 7.

**Table 7:** Comparison of the laccase-mediated degradation of Malachite green by *S. maltophilia* E1 (this study) and other microorganisms reported in the literature

Bacterial strain	Specific conditions	Percentage degradation	By-products	References
*S. maltophilia* E1	Dye concentration—500 ppm, incubation time—24 h	91.8 ± 2%	Michler’s ketone Bis(4-(dimethylamino) phenyl) methanone, 4-(dimethylamino) benzophenone, dibenzylmethane, 4-(dimethylamino) benzaldehyde, and 4-(dimethylamino) phenol	Present study
*Trametes*sp.	Dye concentration—1000 ppm	88.1%	–	Thoa et al. (2022)
*Enterobacter asburiae* strain XJUHX-4TM	Dye concentration—100 ppm; incubation time—72 h, temperature—37 ℃, pH—7.0	80%	Leucomalachite green, desmethyl leucomalachite green, didesmethyl leucomalachite green, (dimethyl amino phenyl)-phenyl methanone, (methyl amino phenyl)-phenyl methanone, (amino phenyl)-phenyl methanone and aniline	Mukherjee and Das (2014)
*Phanerochaete chrysosporium*	Dye concentration—5 ppm, incubation time—24 h, temperature—30 ℃	99%	Leucomalachite green, (amino-phenyl)-phenyl-methanone, di-benzyl methane and 4-(dimethylamino) benzaldehyde	Ghobadi Nejad et al. (2019)
*Trichoderma asperellum*	Dye concentration—122.66 ppm, incubation time—98.58 min, temperature—30 ℃	97.18%	Michler's ketone,4-(dimethylamino) benzophenone, 4-aminobenzophenone and 4-(dimethylamino) benzaldehyde	Shanmugam et al. (2017)
*Bacillus aestuarii* KSK	Dye concentration—100 ppm, incubation time—60 h, temperature— 35 °C, pH—7.0	89%	–	Selvam et al. (2022)
*Trametes* sp.	Dye concentration—50 ppm, temperature—37 ℃	97%	–	Maalej-Kammoun et al. (2009)
*Brevibacillus laterosporus* MTCC 2298	Dye concentration—0.1 ppm, incubation time, 3 h, temperature—30 ℃	87%	Tetradesmethyl leucomalachite green and [4-(1-cyclohexyl)-(1’-phenyl)-methyl]-2, 4-hexenoic acid	Gomare et al. (2009)

However, to check the toxicity of generated metabolites is an important aspect. The phytotoxicity result revealed that laccase treated Malachite green have higher germination percentage, seedling length and vigor compared to untreated Malachite green. This suggested that laccase mediated degradation reduces the toxicity of Malachite green. However, when compared it with control, slight difference was observed in all three parameters (i.e., germination percentage, seedling length and vigor). Similar to our phytotoxicity assay result, laccase mediated Malachite green degradation reduces the toxicity by improving root elongation rate and seed germination of *Phaseolus mungo* and *Triticum aestivum* (Parshetti et al. 2006), *Nicotina tabacum* and *Lactuca sativa* (Yang et al. 2015). According to Du et al. (2011), Malachite green degradation by *Pseudomonas* sp. has been shown to compete and partially eliminate germination inhibition for *Medicago sativa* and *Brassica chinensis*.

Overall, results of the present study revealed that *S. maltophilia* E1 laccase exhibits a capability to degrade Malachite green dye without the requirement of a mediator similarly like other studies (Ghobadi Nejad et al. 2019; Yang et al. 2015), despite the generally lower redox potentials associated with bacterial laccases compared to fungal laccases (Margot et al. 2013). For this, one possibility is that *S. maltophilia* E1 laccase can directly interact with the dye molecules and transfer electrons to the dye molecules without the need for a mediator. This would suggest a high affinity between the laccase active site and the dye molecules, enabling efficient electron transfer (Arregui et al. 2019). Second, the microenvironment surrounding the enzyme within the bacterial cell may provide conditions conducive to mediator-free reactions. This could include factors such as pH, temperature, presence of specific cofactors or molecules within the cell that enhance the performance of enzyme (Bolivar and Nidetzky 2019). Third, *S. maltophilia* laccase might utilize alternative electron transfer pathways or cofactors that compensate for its lower redox potential, thereby enabling it to oxidize dyes efficiently without external mediators (Jones and Solomon 2015). Moreover, the structural characteristics of *S. maltophilia* laccase may differ from typical bacterial laccases, allowing it to interact with dye molecules more effectively. However, to provide a more comprehensive understanding of this phenomenon, further experiments will be needed to conduct in-detail.

## Conclusion

In summary, laccase from *S. maltophilia* E1 was optimized, characterized, and applied for dye decolorization and degradation study. The agro-waste coconut husk emerged as potent substrate for laccase production. The optimized media parameters by BBD improved laccase production three times more than initial unoptimized media. The preferred pH and temperature for crude laccase were 4.0 and 35 ℃, respectively. A crude laccase was also demonstrated to be effective in decolorizing some industrial dyes. In which, laccase effectively decolorized and detoxify Malachite green. Moreover, phytotoxicity data revealed that generated metabolites were less toxic compared to untreated Malachite green. Thus, laccase of *S. maltophilia* E1 will be a promising enzyme for the removal of Malachite green as well as bioremediation of other dye effluents.

## Data Availability

All data generated or analyzed during this study are included in this article.
